# Control Level of Type 2 Diabetes Mellitus in the Elderly Is Associated with Polypharmacy, Accompanied Comorbidities, and Various Increased Risks According to the Beers Criteria

**DOI:** 10.3390/diagnostics13223433

**Published:** 2023-11-13

**Authors:** Burcin Meryem Atak Tel, Gulali Aktas, Satilmis Bilgin, Sumeyye Buse Baltaci, Tuba Taslamacioglu Duman

**Affiliations:** Department of Internal Medicine, Abant Izzet Baysal University Hospital, 14280 Bolu, Turkey; burcinatak@hotmail.com (B.M.A.T.); drsatilmisbilgin@gmail.com (S.B.); busebalcii@hotmail.com (S.B.B.); doktortuuba@gmail.com (T.T.D.)

**Keywords:** type 2 diabetes mellitus, Beers Criteria, polypharmacy, potentially inappropriate medications, HbA1c

## Abstract

Comorbidity rates in the geriatric population have increased because of rising life expectancy; thus, patients have had to use more medications. Type 2 diabetes mellitus, one of the most common diseases, may influence the number of drugs used in geriatric patients. The present study was designed to investigate the association between the level of type 2 DM and polypharmacy. Fifty patients with type 2 diabetes over the age of 65 were included according to the inclusion criteria; 23 were well-controlled and 27 had poorly controlled diabetes. The groups were similar in terms of age, sex, WBC, Hb, Plt, AST, ALT, serum creatinine, fasting glucose, and eGFR levels. Patients with HbA1c values above 7.5 were classified as poorly controlled diabetes patients, and those below were considered well-controlled diabetes patients and were evaluated for inappropriate medication use. The number of medications used daily by the cases (*p* < 0.001), the number of concomitant diseases (*p* = 0.001), and the number of increased risks according to the Beers Criteria (*p* = 0.02) were observed to be high in poorly controlled type 2 diabetes mellitus subjects. HbA1c levels were related to the number of medications (r = 0.4, *p* = 0.004), comorbidities (r = 0.28, *p* = 0.04), and the number of increased risks according to the Beers Criteria (r = 0.31, *p* = 0.014). In conclusion, the number of medications used in patients with poorly controlled type 2 diabetes mellitus was found to be more elevated than in individuals with well-controlled type 2 diabetes mellitus. The HbA1c values varied among patients regarding polypharmacy, comorbidities, and increased risks according to the Beers Criteria.

## 1. Introduction

Type 2 diabetes mellitus constitutes a major health issue globally. Considerable disease burden and mortality are associated with type 2 diabetes mellitus and its complications. Reduced complications have been noted in diabetic patients with the well-controlled disease [1]. Therefore, the factors related to a good control of type 2 diabetes mellitus should be established precisely.

Diabetes mellitus, a chronic metabolic disorder characterized by elevated blood sugar levels, presents unique challenges in the elderly population. As individuals age, their bodies undergo various physiological changes that can complicate the management of diabetes. With the world’s aging population on the rise, the importance of effective diabetic control in elderly patients has never been more critical. This demographic faces an increased risk of complications associated with diabetes, including cardiovascular diseases, neuropathy, kidney dysfunction, and vision impairment. Therefore, a tailored and comprehensive approach to diabetes management in the elderly is essential to improve their quality of life, reduce the risk of complications, and promote overall well-being. In this exploration, we will delve into the multifaceted aspects of diabetic control in elderly diabetic patients, examining the unique considerations, challenges, and strategies that healthcare professionals employ to ensure optimal care for this vulnerable group. Elderly individuals, including diabetics, are prone to have multiple comorbidities. Therefore, prescribing more than one medicine is expected in the geriatric population. The American Geriatrics Society installed the Beers Criteria to detect potentially inappropriate medications in the elderly [2]. The number of increased risks according to the Beers Criteria has been reported to be correlated with the number of medicines used by patients [3].

In the present study, we aimed to observe the factors associated with poor disease control and inappropriate medicine usage in well and poorly controlled diabetic subjects of geriatric age.

## 2. Materials and Methods

**Figure 1:**
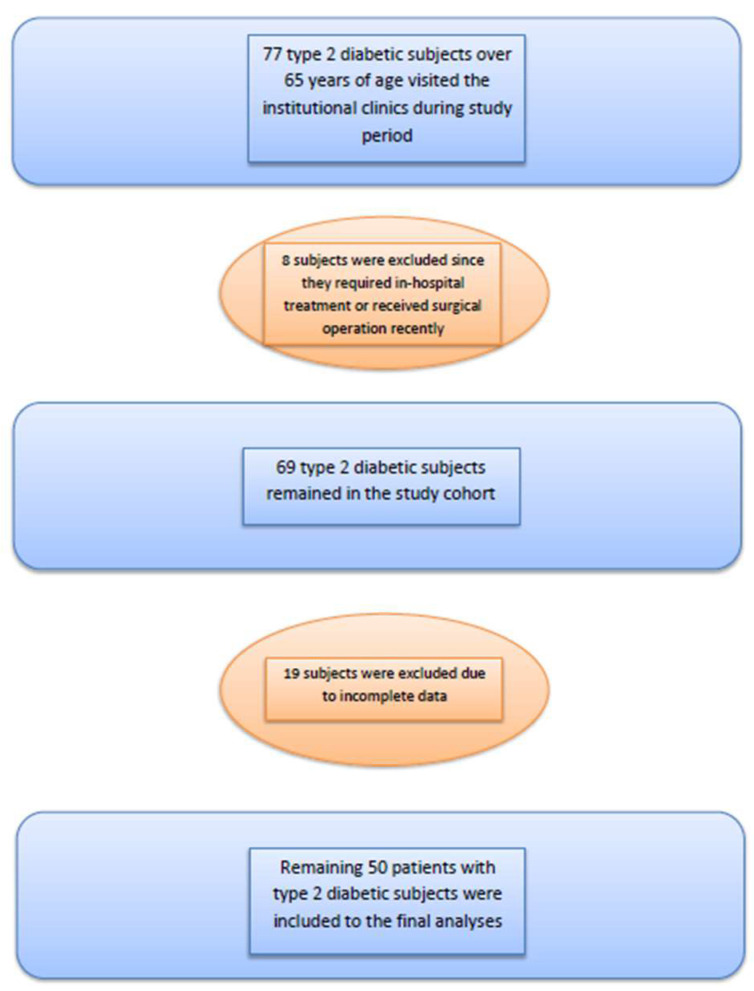
Flow chart of the study.

The institutional database and patients’ files were retrospectively analyzed as soon as approval from the local ethics committee was obtained (approval date: 22-11-2022, approval number: 2022/298). Patients with type 2 diabetes mellitus over 65 years of age were selected from patients who visited outpatient internal medicine clinics of our institution, between November and December 2022. Subjects who required inpatient care in the hospital and who underwent a surgical procedure within the last three months were not included in the study. Patients with incomplete data were also excluded. Figure 1 shows the flow chart of the study.

Age, sex, daily medication usage, and accompanying diseases (for example, chronic obstructive pulmonary disease, hypertension, osteoporosis, heart failure, chronic kidney disease, and neurological disorders) were recorded. Laboratory parameters, including a leukocyte count (WBC), hemoglobin (Hb), a platelet count (Plt), serum creatinine, fasting glucose, glycated hemoglobin (HbA1c), alanine and aspartate transaminases (ALT, AST), and an estimated glomerular filtration rate (eGFR) were noted. The patients were grouped as well or poorly controlled type 2 diabetes mellitus according to the HbA1c level (well-controlled type 2 diabetes mellitus had an HbA1c lower than 7.5%, while poorly controlled type 2 diabetes mellitus had an HbA1c of 7.5% or higher). The threshold of good control in terms of HbA1c level was set at 7.5% when considering the study population’s advanced age.

Potentially inappropriate medications and interactions were defined according to the Beers Criteria [2]. The increased risk of hypoglycemia, failure to assess blood levels, increased risk of hyperkalemia, lack of data on the safety and efficacy of drugs, and increased risks of neurological side effects, bleeding, falls, renal failure, and gastrointestinal side effects were defined as the hazards of potentially inappropriate medication use according to the Beers Criteria. The clinical and laboratory parameters of elderly women and men participants were compared.

Statistics software (SPSS 15.0 for Windows, IBM Co., Chicago, IL, USA) was used to analyze the results. A normality analysis of the study variables was conducted with the Kolmogorov–Smirnov test. A comparison of the variables with and without a normal distribution between the study groups was held by the independent samples *t*-test and Mann–Whitney U test, respectively. The variables with a normal distribution were expressed as the mean ± standard deviation, while the variables with a skewed distribution were shown as the median (min-max). A comparison of the categorical variables was carried out with the X2 test and expressed as numbers and rates. The correlation between the study parameters was conducted with a Pearson’s correlation analysis test. The sensitivity and specificity of the study variables in detecting poor diabetes control were analyzed with receiver operative characteristics (ROC) analysis. The Youden index was used to determine the best cut-off values in ROC analyses. When the *p*-value was lower than 0.05, it was considered statistically significant.

## 3. Results

Fifty patients were enrolled in the study after excluding subjects according to the exclusion criteria. Of those, 23 were in the well-controlled and 27 were in the poorly controlled type 2 diabetes mellitus group. The mean ages of the well and poorly controlled type 2 diabetes mellitus groups were 78 ± 6 years and 76 ±6 years, respectively (*p* = 0.23). Twelve (52%) patients in the well-controlled type 2 diabetes mellitus and 15 (56%) subjects in the poorly controlled type 2 diabetes mellitus group were female. Gender was not statistically different between the study groups (*p* = 0.81).

In the well-controlled T2DM group, 6 did not receive oral anti-diabetic drugs and 14 received metformin alone or in combination with other oral anti-diabetics. One patient received the dipeptidyl peptidase-4 inhibitor and two received sulphonylurea alone in this group. In the poorly controlled T2DM group, 20 subjects were on metformin treatment (either alone or in combination with other drugs) and 2 were on sulphonylurea single drug treatment. Five patients in this group did not receive any oral antidiabetic drugs. Oral anti-diabetic treatment was not statistically different between the study groups (*p* = 0.91). Eleven patients in the well-controlled T2DM group were not on insulin treatment while 12 were on either basal or intensive insulin therapy. In the poorly controlled T2DM group, 17 subjects were on insulin treatment (basal or intensive) and 10 did not receive insulin treatment. The insulin treatment rate was not significantly different between the well and poorly controlled T2DM groups (*p* = 0.74).

The WBCs of the well and poorly controlled diabetic elderly patients were 7.9 ± 2.3 k/mm^3^ and 7.7 ± 1.9 k/mm^3^, respectively (*p* = 0.76). The mean Hbs of the well and poorly controlled type 2 diabetes mellitus groups were 12.2 ± 1.9 g/dL and 12.4 ± 1.5 g/dL, respectively (*p* = 0.58). The mean platelet value of the well-controlled diabetic elderly patients (283 ± 98 k/mm^3^) was not statistically different from the mean platelet value of the poorly controlled diabetic patients (271 ± 93 k/mm^3^), (*p* = 0.67). The median aspartate transaminases of the well and poorly controlled type 2 diabetes mellitus groups were 16 (8–40) U/L and 15 (7–38) U/L, respectively (*p* = 0.3). Similarly, the median alanine transaminase level of the well-controlled type 2 diabetes mellitus group (14 (8–30) U/L) was not significantly different from that of the poorly controlled type 2 diabetes mellitus group (13 (7–34) U/L), (*p* = 0.37). The fasting plasma glucose of the well and poorly controlled diabetic subjects was 119 (96–168) mg/dL and 121 (91–234) mg/dL, respectively (*p* = 0.52). The serum creatinine of the well-controlled type 2 diabetes mellitus patients (1 (0.74–2.7) mg/dL) was not statistically different from that of the poorly controlled elderly type 2 diabetes mellitus subjects (0.99 (0.69–2,3) mg/dL), (*p* = 0.42). The blood ureas of the well and poorly controlled type 2 diabetes mellitus patients were 67 (15–88) mg/dL and 64 (26–91) mg/dL, respectively (*p* = 0.5). The median HbA1c level of the well-controlled type 2 diabetes mellitus patients (6.9 (5.8–7.45)%) was significantly reduced compared to the HbA1c level of the poorly controlled elderly type 2 diabetes mellitus patients (8.9 (7.67–13)%). The difference between the groups was statistically significant (*p* < 0.001). The data of the well and poorly controlled type 2 diabetes mellitus groups were summarized in Table 1.

The number of daily medicines used was four (2–9) in elderly patients with well-controlled type 2 diabetes mellitus and eight (4–13) in subjects with poorly controlled diabetes mellitus. The difference between the well and poorly controlled diabetes mellitus patients in terms of the daily number of medicines was statistically significant (*p* < 0.001).

The number of comorbidities in the well and poorly controlled diabetes mellitus patients was two (1–6) and four (1–7), respectively. The difference in the comorbidities between the well and poorly controlled elderly diabetes mellitus patients was statistically significant (*p* = 0.001).

The number of increased risks according to the Beers Criteria in elderly subjects with well-controlled type 2 diabetes mellitus was one (0–2), and in subjects with poorly controlled type 2 diabetes mellitus was two (0–6). The difference between the groups was statistically significant (*p* = 0.02). Table 2 shows the daily medicine, comorbidities, and increased risks of the well and poorly controlled type 2 diabetes mellitus groups.

Increased hyperkalemia (*p* = 0.016) and increased bleeding risks (*p* = 0.038) were found more frequently in the poorly controlled subjects compared to the well-controlled type 2 diabetes mellitus group. Other increased risks were similar among the study groups (*p* > 0.05 for all).

All patients except six (26.1%) received oral hypoglycemic drugs in the well-controlled diabetes group, while all subjects except five (18.5%) received oral hypoglycemic drugs in the poorly controlled group (*p* = 0.95). Only 11 (48%) subjects in the well-controlled diabetes group and 10 (37%) subjects in the poorly controlled group were not receiving insulin treatment (*p* = 0.74). The rate of increased hypoglycemia risk was 21.7% (5 out of 23 subjects) in the well-controlled group and 33.3% (9 out of 27 subjects) in poorly controlled elderly patients with type 2 diabetes mellitus (*p* = 0.36).

One (4.3%) in the well-controlled group and two (7.4%) in the poorly controlled group had no additional comorbidities other than type 2 diabetes mellitus (*p* = 0.65). Table 3 shows the comorbidity rates of the study cohort.

Pearson’s correlation analysis revealed that the HbA1c levels were significantly and positively correlated with the number of medicines (r = 0.4, *p* = 0.004), the number of comorbidities (r = 0.28, *p* = 0.04), and the number of increased risks according to the Beers criteria (r = 0.31, *p* = 0.014).

**Figure 2:**
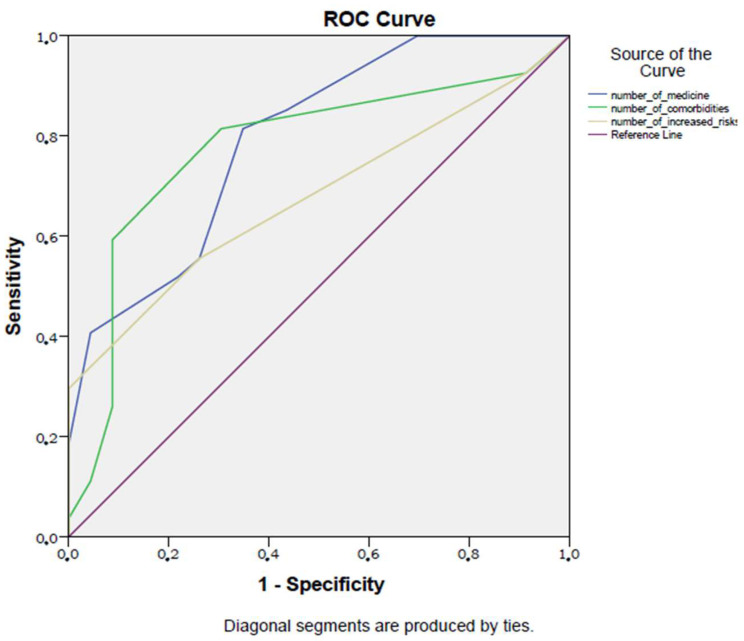
ROC curve of study parameters in detecting poor diabetic control.

In ROC analysis, the sensitivity and specificity of the number of daily medicines used (when five or more) in detecting poor diabetic control were 85% and 56%, respectively (AUC: 0.8, *p* < 0.001, 95% CI: 0.67–0.92). The sensitivity and specificity of the accompanied comorbidities (when there were three or more) in detecting poor diabetes control were 82% and 70%, respectively (AUC: 0.78, *p* = 0.001, 95% CI: 0.64–0.92). The sensitivity and specificity of the number of increased risks according to the Beers Criteria (when there were two or more increased risks) in detecting poorly controlled type 2 diabetes mellitus in the elderly were 57% and 74%, respectively (AUC: 0.68, *p* = 0.03, 95% CI: 0.53–0.83). Figure 2 shows the ROC curves of these parameters in detecting poor diabetic control in the study population.

## 4. Discussion

The main findings of the present study are as follows: (a) the number of daily medicines of the patients, the number of accompanying diseases, and the number of increased risks according to the Beer criteria are higher in poorly controlled diabetic subjects compared to the well-controlled diabetic patients; (b) hyperkalemia and bleeding risks are higher in poorly controlled diabetics compared to the well-controlled subjects; (c) HbA1c was positively correlated with both of the numbers of medicines, comorbidities, and increased risks according to the Beers Criteria; and (d) the number of medicines has the highest sensitivity, and the number of increased risks has the highest specificity in selecting diabetic subjects with poor control.

Comorbidities generally raise the risk of side effect development primarily due to the increased number of drugs used by patients who have multiple accompanying diseases [4]. On the other hand, physicians may be unwilling to prescribe necessary medicines to the elderly since the concern of polypharmacy and multiple comorbidities usually present in this population [5]. However, while prescribing appropriate and necessary treatment agents, physicians could eliminate polypharmacy by eliminating redundant and ineffective therapies [6]. The Beers Criteria should be consulted when a drug is considered for the elderly, especially for patients with type 2 diabetes mellitus [7]. Therefore, we studied increased risks according to the Beers Criteria in the type 2 diabetic population in the present study. A higher rate of increased risks in the poorly controlled subjects may be a consequence of physicians trying to prevent diseases with more drugs.

Multimorbidity is defined by the presence of multiple chronic conditions [8]. The number of multimorbid elderly individuals is rising year by year, mainly due to the increased life expectancy and the advances in medicine in treating chronic conditions with high mortality rates. Multimorbidity causes polypharmacy in this population, and in turn, inappropriate medications and adverse drug reactions become inevitable [9]. Authors suggest better hypertension control with achieving sustained blood pressure goals for the sake of reducing comorbidities [10]. However, better blood pressure control usually requires combined antihypertensive treatment [11], which in turn contributes to polypharmacy. A recent study showed that advancing age and accompanying morbidities make older people vulnerable to polypharmacy and the use of inappropriate drugs concomitantly [12]. Another report revealed that the mean number of medicines in older adults was 5.9 [13]. This number is higher than what we reported in the patients with well-controlled type 2 diabetes mellitus and lower than in the patients with poorly controlled type 2 diabetes mellitus. It is probably because of the increased number of accompanying diseases in this group, which necessitates additional prescriptions of other medicines to manage comorbidities or alleviate symptoms, such as pain.

Older individuals usually use more than one medication, and type 2 diabetes mellitus may even increase the number of drugs used daily in this population. Polypharmacy brings some risks with it. The hospital admissions of older subjects are elevated by polypharmacy [14]. McCracken et al. declared that elderly diabetics who use more medications were usually over-treated [15]. Similarly, Huang et al. noted that the prevalence of polypharmacy was higher in diabetic older adults compared to the non-diabetic age-matched controls [16]. Using combination therapies in diabetic subjects is not always associated with better disease control. Indeed, a Brazilian study found that polypharmacy in elderly diabetics was not related to a good control of type 2 diabetes mellitus [17]. Following the literature data, the results of our present work pointed out that the number of medications used was higher in patients with poorly controlled ones than in the subjects with well-controlled type 2 diabetes mellitus.

Polypharmacy, the simultaneous use of multiple medications by an individual, is a growing concern in modern healthcare, particularly among individuals with type 2 diabetes mellitus [18]. This chronic metabolic disorder, characterized by insulin resistance and elevated blood glucose levels, often necessitates the use of various medications to manage the condition effectively. However, as the number of prescribed drugs increases, so does the complexity of medication regimens, leading to potential challenges in disease control and overall patient well-being [19]. Type 2 diabetes mellitus is a prevalent condition that affects millions of individuals worldwide, and its management frequently involves a combination of lifestyle modifications and pharmacological interventions. While these medications can be instrumental in achieving glycemic control and reducing the risk of complications, they also introduce the risk of polypharmacy, which can have unintended consequences such as drug interactions, adverse effects, reduced medication adherence, and increased healthcare costs. A cross-sectional study by Dobrica et al. revealed that diabetic patients had more comorbidities and received more drugs than control subjects, which led to a higher rate of drug–drug and drug–food interactions in the diabetic cohort [20]. Similarly, a study from Portugal analyzed inappropriate medicine use in patients with type 2 diabetes mellitus and reported that drug–drug interactions and potentially inappropriate medicines were more common in diabetic subjects with polypharmacy [21]. On the other hand, it is well established that the increased number of medications used by the elderly increases the risk of drug-related side effects [22]. In contrast, in another study involving more than 3000 individuals over 65 years of age, drug-related side effects were not significantly higher in diabetic patients compared to the non-diabetic age-matched controls [23]. Adherence to the treatment is another issue in diabetic elderly with polypharmacy. Polypharmacy significantly reduced the treatment adherence of diabetic subjects in a study from the United States [24]. However, a recent study found that the number of medications was not associated with treatment adherence in diabetic elderly [25]. Medications increase the healthcare cost of the diabetic subjects [26]. Accordingly, attempts to prevent polypharmacy in the diabetic population significantly reduced the healthcare costs according to a study from Japan [27]. Treatment non-adherence not only prevents diabetic patients from reaching target blood sugar levels, but may also lead physicians to prescribe more medications to ensure diabetic regulation. In accordance with the literature data, we found that poorly controlled diabetes was associated with a higher number of daily drug use compared to the well-controlled diabetic elderly. Moreover, probably due to polypharmacy, the rate of inappropriate medication use according to the Beers Criteria was higher in poorly controlled subjects than in the elderly patients with well controlled type 2 diabetes mellitus.

Type 2 diabetes mellitus, a chronic metabolic condition characterized by insulin resistance and high blood sugar levels, is often not an isolated health concern. Many individuals living with type 2 diabetes also contend with a complex web of comorbid conditions, which can significantly impact their overall health and complicate the management of diabetes itself [28]. These comorbid diseases, ranging from cardiovascular disorders and hypertension to kidney disease and obesity, create a unique challenge for healthcare professionals tasked with achieving effective disease control in individuals with type 2 diabetes. The presence of comorbid diseases in diabetes patients introduces a multifaceted clinical landscape, where the interplay between conditions necessitates a tailored and holistic approach to care [29]. Managing one condition can influence the course of another, and the medications used to control one ailment may have implications for glycemic control and vice versa. As the prevalence of type 2 diabetes continues to rise globally, understanding and addressing the intricate relationship between comorbid diseases and disease control in diabetes becomes increasingly vital for optimizing patient outcomes and enhancing their quality of life. In a study with a large cohort (more than 900,000 individuals), authors reported that accompanied chronic conditions were associated with poor glycemic control in patients with type 2 diabetes mellitus [30]. Similarly, a meta-analysis that includes more than 21,000 participants revealed that poor glycemic control in type 2 diabetes mellitus was associated with the presence of comorbidities [31]. In line with the literature knowledge, we found a higher rate of comorbidity in poorly controlled diabetic elderly patients compared to the individuals with well-controlled disease.

The correlation between diabetic control and the number of daily medicines used is a complex and multifaceted relationship influenced by several interrelated factors. Here are some key reasons that help explain this correlation: Disease Complexity; type 2 diabetes mellitus is a multifactorial condition influenced by genetics, lifestyle, and various metabolic factors [32]. As the disease progresses, it often becomes more complex, requiring a combination of medications to address the different underlying mechanisms. The need for multiple medications increases as type 2 diabetes mellitus becomes more severe [33]. Individual Variability; people with diabetes vary in terms of their disease progression and response to treatment. Some individuals may achieve adequate glycemic control with lifestyle modifications or a single medication, while others may require a combination of drugs to manage their blood sugar effectively [34]. Comorbid Conditions; many individuals with type 2 diabetes have comorbid conditions such as hypertension, dyslipidemia, obesity, or cardiovascular disease [35]. These conditions often require additional medications for management. As a result, the number of prescribed medications increases, and the potential for polypharmacy, or the taking of multiple drugs, rises. Medication Progression; diabetes management typically follows a stepwise approach [36]. Healthcare providers start with lifestyle modifications and one or two oral medications, but over time, as blood sugar levels become more challenging to control, additional medications, such as insulin or injectable drugs, may be required. Targeting Different Mechanisms; diabetes medications work through various mechanisms, such as improving insulin sensitivity, increasing insulin secretion, reducing glucose production by the liver, or slowing down carbohydrate absorption [37]. Using multiple medications allows healthcare providers to target different aspects of the disease and enhance glycemic control. Individualized Treatment; diabetes management is highly individualized. Healthcare providers tailor treatment plans based on a patient’s specific needs, preferences, and response to medications [38]. Some patients may require more medications than others to achieve target blood sugar levels. Risk Factors and Complications; diabetes increases the risk of complications such as diabetic nephropathy, neuropathy, and cardiovascular disease [39]. Managing these complications often involves additional medications to control blood pressure, cholesterol levels, and prevent further damage to organs. Adherence Challenges; as the number of daily medications increases, the potential for medication non-adherence also rises [40]. Patients may struggle with the complexity of their medication regimens, which can negatively impact diabetic control. Potential for Drug Interactions; with multiple medications, there is an increased risk of drug interactions, which can affect the effectiveness and safety of the treatment [21]. Healthcare providers need to carefully consider potential interactions when prescribing multiple drugs. The correlation between diabetic control and the number of daily medicines used is a reflection of the complexity of diabetes as a disease and the need to address various aspects of its management. Tailored treatment plans, regular monitoring, and patient education are essential to ensure that the benefits of multiple medications outweigh the potential risks associated with polypharmacy and contribute to effective diabetic control.

The Beers Criteria, developed by the American Geriatrics Society, is a set of guidelines that identifies medications that are potentially inappropriate for use in older adults due to their increased risk of adverse effects or drug interactions [2]. The correlation between diabetic control and the increased risks identified in the Beers Criteria can be explained by several factors. One of these factors could be polypharmacy. As individuals with diabetes age, they are more likely to develop comorbid conditions, such as hypertension, heart disease, and kidney problems, which may require additional medications. This can lead to polypharmacy, the concurrent use of multiple medications, which is a known risk factor for medication-related problems [22]. Polypharmacy can increase the chances of drug interactions, medication errors, and adverse effects, all of which can negatively impact diabetic control. Another factor is the side effects of the medications. Some medications listed in the Beers Criteria may have side effects that can affect blood glucose levels or exacerbate diabetic symptoms. For example, certain antipsychotic medications can cause weight gain, which is detrimental to diabetic control, as excess weight can worsen insulin resistance [41]. The third factor is the risk of hypoglycemia. Some medications, such as insulin and sulfonylureas, which are used to treat diabetes can increase the risk of hypoglycemia [42]. When these medications are combined with other drugs that affect blood glucose or increase the risk of hypoglycemia (e.g., certain antihypertensive medications or antidepressants), it can lead to more frequent and severe episodes of hypoglycemia, posing a risk to diabetic control. The fourth factor could be impaired renal function. Many older adults with diabetes also have reduced kidney function, a common comorbidity in this population [43]. Some medications listed in the Beers Criteria are known to be nephrotoxic (harmful to the kidneys) or require dose adjustments in individuals with impaired renal function. These medications can affect the clearance of other drugs from the body, potentially leading to drug accumulation and adverse effects, which can complicate diabetic control. We can consider cognitive impairment as the fifth factor. Some medications listed in the Beers Criteria may have central nervous system (CNS) depressant effects, leading to cognitive impairment or sedation. In individuals with type 2 diabetes mellitus, especially older adults, cognitive impairment can hinder their ability to manage their disease effectively, monitor blood glucose levels, and adhere to their treatment plan [44]. Another factor that contributes to the correlation between the Beers Criteria and diabetic control is orthostatic hypotension. Certain medications can cause orthostatic hypotension, a sudden drop in blood pressure upon standing. This can increase the risk of falls and injuries in older adults with type 2 diabetes mellitus, potentially leading to non-adherence to their diabetic control regimen [45]. The seventh and last factor is gastrointestinal symptoms in the elderly population. Some medications may cause gastrointestinal side effects, such as nausea, diarrhea, or constipation [46]. These symptoms can interfere with dietary choices and meal planning, making it more challenging for individuals to manage their diabetes through proper nutrition. Hence, the correlation between diabetic control and the increased risks identified in the Beers Criteria is rooted in the complex interactions between the medications used to manage diabetes and those used to treat other health conditions in older adults. Healthcare providers must carefully consider the potential risks and benefits of each medication when designing treatment plans for older adults with diabetes to optimize diabetic control while minimizing the risks associated with medication use. Regular monitoring and medication reviews are essential to ensure the safety and effectiveness of the treatment regimen. In the present work, we reported that increased risks according to the Beers Criteria were more common in patients with poor diabetic control. Controlling and reducing those risks may have beneficial effects on reaching blood glucose targets in the elderly population with type 2 diabetes mellitus.

There are several limitations of the present study. One of them is the relatively small study population which could decrease the importance of the study results. The other is the single-center nature of the study design which could make it difficult to globalize the study results. Moreover, 27 subjects were excluded from the present study, which can be considered as selection bias. However, a remarkable correlation between HbA1c and polypharmacy, multiple comorbidities, and increased risks according to the Beers criteria may add significantly to the current medical literature.

## 5. Conclusions

In conclusion, according to the Beers Criteria, increased medications, comorbidities, and risks are associated with poor disease control in elderly subjects with type 2 diabetes mellitus. Physicians should be aware of this situation and attempt to reduce these risks by reviewing the medications of the elderly to achieve better diabetic control.

## Figures and Tables

**Table 1:** Data of well and poorly controlled T2DM patients.

		Well-Controlled T2DM (*n* = 23)	Poorly Controlled T2DM (*n* = 27)	*p*
Sex	Female (*n*, (%))	12 (52)	15 (56)	0.81
	Male (*n*, (%))	11 (48)	12 (44)	
		*Mean ± SD*		
Age (years)		78 ± 6	78 ± 6	0.23
WBC (k/mm^3^)		7.9 ± 2.3	7.7 ± 1.9	0.76
Hb (g/dL)		12.2 ± 1.9	12.4 ± 1.5	0.58
Plt (k/mm^3^)		283 ± 98	271 ± 93	0.67
		*Median (min.-max.)*		
AST (U/L)		16 (8–40)	15 (7–38)	0.3
ALT (U/L		14 (8–30)	13 (7–34)	0.37
HbA1c (%)		6.9 (5.8–7.45)	8.9 (7.67–13)	<0.001
Creatinine (mg/dL)		1 (0.74–2.7)	0.99 (0.69–2.3)	0.42
eGFR (%)		67 (15–88)	64 (26–91)	0.5
Fasting glucose (mg/dL)		119 (96–168)	121 (91–234)	0.52

**Table 2:** Daily medicine, comorbidities, and increased risk numbers of the study population.

	Well-Controlled T2DM (*n* = 23)	Poorly Controlled T2DM (*n* = 27)	*p*
Number of daily medicines (*n*)	4 (2–9)	8 (4–13)	<0.001
Number of comorbidities (*n*)	2 (1–6)	4 (1–7)	0.001
Number of increased risks according to the Beers Criteria (*n*)	1 (0–2)	2 (0–6)	0.02

**Table 3:** Comorbidity rates of the study group.

	Well-Controlled T2DM (*n* = 23)	Poorly Controlled T2DM (*n* = 27)	*p*
Comorbidity (*n* (%))	1 (4.7%)	2 (7.4%)	0.65

## Data Availability

Data regarding this work are available from the corresponding author upon reasonable request.
